# Characterization of Volatile Compounds in Four Different *Rhododendron* Flowers by GC×GC-QTOFMS

**DOI:** 10.3390/molecules24183327

**Published:** 2019-09-12

**Authors:** Chen-Yu Qian, Wen-Xuan Quan, Zhang-Min Xiang, Chao-Chan Li

**Affiliations:** 1 Guangdong Provincial Key Laboratory of Emergency Test for Dangerous Chemicals/Guangdong Engineering and Technology Research Center for Ambient Mass Spectrometry, Guangdong Institute of Analysis, Guangzhou 510070, China; qianchenyu94@163.com; 2 Guizhou Provincial Key Laboratory of Mountainous Environmental Protection, Guizhou Normal University, Guiyang 550001, China; wenxuanq@gznu.edu.cn

**Keywords:** *Rhododendron* flowers, volatile compounds, comprehensive two-dimensional gas chromatography-mass spectrometry, quadrupole time-of-flight mass spectrometry, odor description

## Abstract

Volatile compounds in flowers of *Rhododendron delavayi*, *R. agastum*, *R. annae*, and *R. irroratum* were analyzed using comprehensive two-dimensional gas chromatography-mass spectrometry (GC×GC) coupled with high-resolution quadrupole time-of-flight mass spectrometry (QTOFMS). A significantly increased number of compounds was separated by GC×GC compared to conventional one-dimensional GC (1DGC), allowing more comprehensive understanding of the volatile composition of *Rhododendron* flowers. In total, 129 volatile compounds were detected and quantified. Among them, hexanal, limonene, benzeneacetaldehyde, 2-nonen-1-ol, phenylethyl alcohol, citronellal, isopulegol, 3,5-dimethoxytoluene, and pyridine are the main compounds with different content levels in all flower samples. 1,2,3-trimethoxy-5-methyl-benzene exhibits significantly higher content in *R. irroratum* compared to in the other three species, while isopulegol is only found in *R. irroratum* and *R. agastum*.

## 1. Introduction

The evergreen woody shrub genus *Rhododendron* is one of the largest genera in the family Ericaceae, and more than 1000 species are currently recognized; of these, 567 species representing 6 subgenera are known from China [1,2]. *Rhododendrons* are not only of high ornamental value but also good medicinal plants. Flowers of *Rhododendron* provide a large number of bioactive natural chemical products, including diterpenoids [3], flavonoids [4], and phenols [5], which are known to be effective for the treatment of rheumatism [6] and to have anti-inflammatory [7], anti-cancer [8], and antioxidant [9] properties. Volatile compounds from flowers also provide some ecological functions [10], including in the role of pollinators [11] and as defenders against nectar-thieving ants [12]. Aside from their ecological functions, flower volatiles have some aesthetic and emotional benefits for humans [13]. On the other hand, different volatile compounds may influence the odor, both in an individual and in a synergistic or antagonistic way, which in turn could be related to one or more chemical compounds or compound classes [14]. In order to investigate the aroma characteristics of *Rhododendron* flowers, it is important to research specific volatile constituents as thoroughly as possible. *Rhododendron irroratum*, *R. delavayi*, *R. annae*, and *R. agastum* are ecologically and horticulturally important alpine flowers and are also the pioneer and constructive species in Baili *Rhododendron* National Forest Reserve in the Guizhou province of China [15]. *R. delavayi* belongs to the subsection Arborea, while *R. irroratum*, *R. annae*, and *R. agastum* belong to the subsection Irrorata. *R. irroratum* is one of the large-flowered *Rhododendron* species [16]. These species were chosen for the investigation of volatile odor constituents in different *Rhododendron* flowers.

Gas chromatography–mass spectrometry (GC-MS) has long been the primary technique used to detect the aroma components of various plants [17,18]. However, GC-MS can only identify a limited number of separable compounds due to its insufficient peak capacity, limited resolved power, and low sensitivity [19]. The combination of gas chromatography with high-resolution quadrupole time-of-flight mass spectrometry (QTOFMS) has been demonstrated for analysis in different fields, including flavor research [20] and volatile profiling [21], and has proved to be a powerful analytical tool. However, the limited chromatographic separation power inevitably causes co-elution problems for complex samples. Compared with traditional one-dimensional gas chromatography (1DGC), comprehensive two-dimensional gas chromatography (GC×GC), which has appeared as a new analytical technique based on the application of two GC columns with different stationary phases, provides substantially enhanced resolving power and peak capacity. GC×GC leads to linear distributions of homologous series in 2D chromatograms, thus greatly reducing the coelution problem [22]. GC×GC can thus be a more suitable tool for analysis of the complex chemical systems of plant aroma, where the number of volatile aroma compounds is large and some of them are present at trace levels [23]. Recently, GC×GC technology has been successfully applied for the assessment of various plants such as teas [24], berries [25], and tobacco [26]. To date, few reports have studied the volatile chemical components in *Rhododendron* flowers by 1DGC. With the 1DGC technique, 9,12,15-octadecatrienoic acid,[Z,Z,Z]-, phytol, and *n*-hexadecanoic acid were found to be the major compounds in flowers of *R. mucronatum* and *R. simii* [27]; while *R. ponticum* comprises mostly α-pinene, β-pinene, and linalool [28], in flowers of *R. schlippenbachii*, only 39 hydrophilic compounds could be detected by 1DGC [29]. A previous study reported the volatile compounds in the leaves, stems, and roots of six *Rhododendron* species [15]. However, the volatile components in flowers of the four *Rhododendron* species in the present study have never been investigated by the GC×GC approach before. Therefore, it is necessary to study the flowers’ volatile compounds in order to explore odor characterizations.

In this study, GC×GC-QTOFMS was used in combination with headspace solid-phase microextraction (HS-SPME) to conduct in-depth analysis of the volatile aroma constituents in different *Rhododendron* flowers. The advantages of GC×GC–QTOFMS were exploited for high-throughput, untargeted chromatographic profiling of complex samples. The volatile compounds and their corresponding contents in various representative *Rhododendron* samples were examined. The obtained results provide useful information for establishing a volatile aroma chemical database from *Rhododendron* flowers.

## 2. Results and Discussion

### 2.1. Comparison of 1DGC and GC×GC

**Figure 1:**
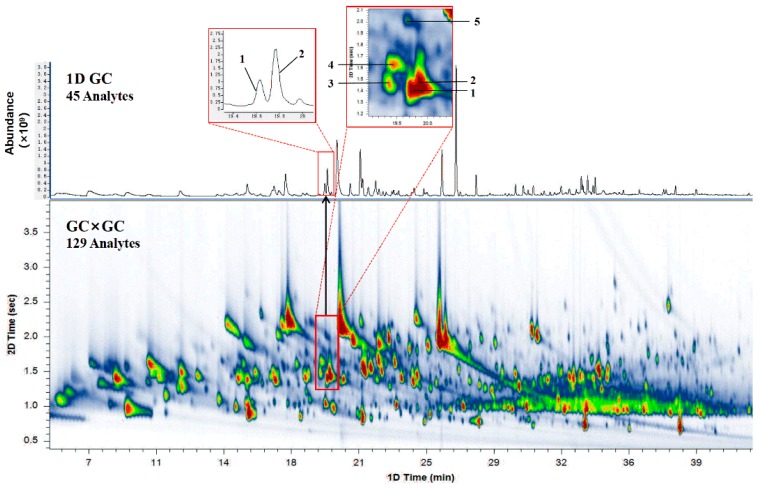
Chromatographic analysis of *Rhododendron* by GC-quadrupole time-of-flight mass spectrometry (QTOFMS) and a comprehensive two-dimensional gas chromatography–mass spectrometry (GC×GC)-QTOFMS color diagram (1: linalool; 2: 2-nonen-1-ol; 3: linalool oxide; 4: *p*-cymenene; 5: benzoic acid, methyl ester).

In typical 1DGC analysis, it is often difficult to achieve pure mass spectra for compounds in a co-elution peak, thus leading to unreliable results. With improved separation power and enhanced sensitivity, the GC×GC technique is able to resolve and detect more volatile aroma compounds in a complex sample compared to conventional one-dimensional GC-MS [30]. A clear illustration demonstrating the employment of GC×GC is presented in Figure 1. Both the chromatogram obtained by GC×GC–TOF/MS using a 4 s modulation period and the total ion chromatography by 1DGC are shown. As can be seen in the partial chromatograms obtained by 1DGC and GC×GC-QTOFMS, linalool (Peak 1, ^1^*t*_R_ = 19.783 min, ^2^*t*_R_ = 1.405 s) and 2-nonen-1-ol (Peak 2, ^1^*t*_R_ = 19.849 min, ^2^*t*_R_ = 1.447 s) were responsible for the two peaks detected between retention times of 19.380 min and 19.850 min on the HP-5 MS column. However, three other minor compounds in addition to these two peaks were further separated as they exhibited different polarities on the DB-17 MS column; these were linalool oxide (Peak 3, ^1^*t*_R_ = 19.383 min, ^2^*t*_R_ = 1.467 s), *p*-cymenene (Peak 4, ^1^*t*_R_ = 19.450 min, ^2^*t*_R_ = 1.627 s), and benzoic acid, methyl ester (Peak 5, ^1^*t*_R_ = 19.716 min, ^2^*t*_R_ = 2.017 s). The co-eluted compounds in the peak region were interfered with by dominating compounds and would usually be ignored due to their low concentrations. In summary, GC×GC successfully resolved a total of 129 compounds, while only 45 compounds were separated in 1DGC (Table S1). The results revealed the great advantages of GC×GC analysis, which is suitable for the investigation of volatile compounds in complex samples.

### 2.2. Identification of Common Volatile Components

GC×GC–QTOFMS was used to characterize the detailed chemical composition of all the samples. Several hundred peaks were generated in the GC×GC contour plot with a peak detection threshold of S/N > 3. In total, 129 volatile compounds were tentatively identified in four *Rhododendron* samples based on (1) spectral similarity (both match and reverse match scores of >750), (2) comparison with molecular ions (within 5 ppm), if they existed, and (3) retention index (RI, ±35). Table S1 lists the complete information of the 129 volatile constituents detected by GC×GC-QTOFMS.

**Figure 2:**
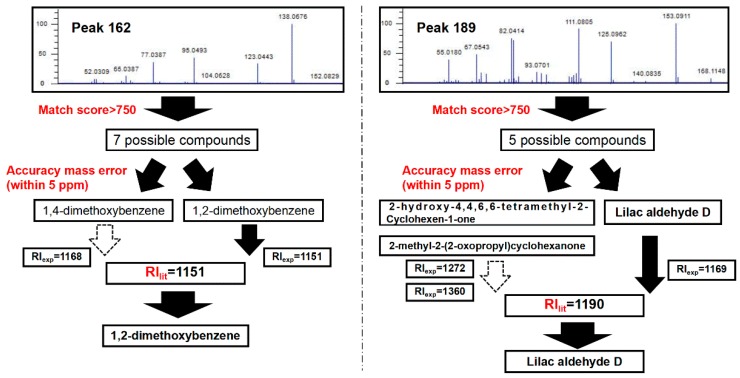
Diagram illustrating the process of compound confirmation in GC×GC-QTOFMS.

Figure 2 introduces the identification process of two examples (1,2-dimethoxybenzene and lilac aldehyde D). First, the National Insititute of Standards and Technology (NIST) library search for Peak 162 and Peak 189 resulted in 7 and 5 possible compounds, respectively, with match factor >750. Then, only exact mass analyses within a mass accuracy of <5 ppm were considered. For Peak 162, the measured accurate mass was 138.0676, which corresponds to a formula of C_8_H_10_O_2_. The accurate mass reduced the number of possible compounds to two isomers (1,2-dimethoxybenzene and 1,4-dimethoxybenzene). Last, their retention indices were reviewed for further confirmation. The GC×GC analysis provided an experimental RI value of 1151 for this peak, which matched 1,2-dimethoxybenzene (literature RI value of 1151) rather than 1,4-dimethoxybenzene (RI_lit_ = 1168). Therefore 1,2-dimethoxybenzene was the final identified compound for Peak 162.

Taking Peak 189 as another example: The NIST library search provided several possible compound matches. Among them, seven possible compounds were screened out according to their relatively high match scores. Subsequently, the mass spectrum provided a measured mass of 168.1148, corresponding to a chemical formula of C_10_H_16_O_2_. This indicated that 2-methyl-2-(2-oxopropyl) cyclohexanone, lilac aldehyde D, and 2-hydroxy-4,4,6,6-tetramethyl-2- cyclohexen-1-one were three possible compounds with the theoretical molecular ion mass of 168.1145. Lastly, the experimental RI value of the peak (RI_exp_ = 1190) confirmed that lilac aldehyde D with RI_lit_ of 1169 was the final identified compound for Peak 189, while the other two candidates, 2-methyl-2-(2-oxopropyl) cyclohexanone (RI_lit_ = 1360) and 2-hydroxy-4,4,6,6-tetramethyl-2-cyclohexen-1-one (RI_lit_ = 1272), were screened out. The results emphasis the importance of applying further confirmation on the base of the spectral library match, since the compound with the highest match factor might be mistaken for the identity of the component [31]. In conclusion, with complementary identification processes, GC×GC coupled with high-resolution QTOFMS produces more precise compound identification results.

### 2.3. Volatile Component Analysis

**Figure 3:**
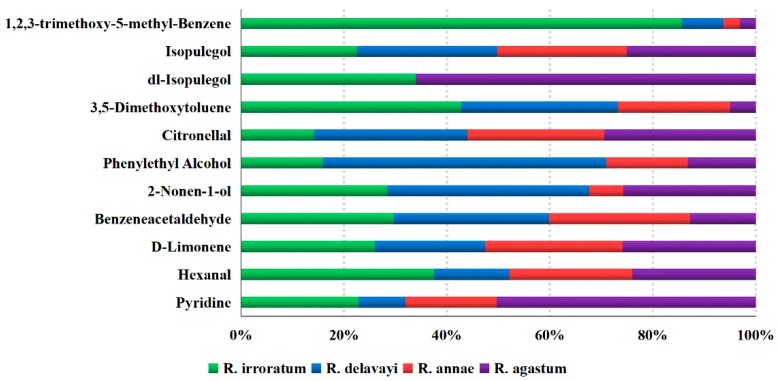
Distribution (%) of major compounds presented in four different species of *Rhododendron*.

In order to establish the experimental conditions, the mixed sample was analyzed via GC×GC-QTOFMS in triplicate. The intraday precision was evaluated by analyzing three equivalent mixed samples on the same day, and this was then repeated for three consecutive days to determine the interday precision. As shown in Table S2, the intraday and interday precision were expressed as the relative standard deviation (RSD). RSD values of no more than 25% were found in each compound in the mixed sample, demonstrating the good repeatability of the GC×GC-QTOFMS method. Subsequently, the four flower species were analyzed by the established method. The relative contents (%) of compounds in each sample were calculated based on the ratio of the area of the corresponding peak to the total peak area; the averages of the relative contents of each compound in the *Rhododendron* flower samples are tabulated in Table S2. Figure 3 presents the distribution (%) of the major compounds in the four different species of *Rhododendron*. Among them, benzeneacetaldehyde was found in all flower species with high content in *R. irroratum* (6.255% ± 0.951%), *R. delavayi* (7.013% ± 0.059%), and *R. annae* (6.349% ± 0.062%), whereas it presented with relatively low content in *R. agastum* (2.987% ± 0.357%). Citronellal presented the highest content in *R. annae* (7.004% ± 0.028%) and *R. agastum* (7.722% ± 0.303%), and was the second most abundant component in *R. delavayi* (7.944% ± 0.225%), but was slightly low in *R. irroratum* (4.178% ± 0.654%). Both benzeneacetaldehyde and citronellal contribute to the sweet floral profile of these samples. Benzeneacetaldehyde has a grassy odor, while citronellal has a slight hyacinth odor. 1,2,3-trimethoxy-5-methyl-benzene was detected in all species with content ranging from trace (0.243% ± 0.023% in *R. annae*) to abundant (6.046% ± 0.623% in *R. irroratum*). On the other hand, isopulegol was detected only in *R. irroratum* and *R. agastum*, with a highest content of 7.722% ± 0.407% in *R. agastum*. Thus, this compound can be used to discriminate *R. agastum* or *R. irroratum* from other *Rhododendron* species. Phenylethyl alcohol accounted for a significantly high content in *R. delavayi* (up to 8.922% ± 0.061%) compared to in the other three species and was characterized as a dried rose floral aroma. Similarly, 2-nonen-1-ol, with a sweet melon odor, also presented the highest content in *R. delavayi* (5.633% ± 0.813%) but slightly low in *R. irroratum* (4.299% ± 0.288%) and *R. agastum* (4.071% ± 0.378%). Limonene and isopulegol presented in all species with relatively low content compared with other major compounds and with no significant differences between species. Limonene has a sweet citrus or orange odor, while isopulegol has a minty or woody odor. Although the rest of the compounds had relatively low threshold values due to their low contents, they all play a certain role in the odor characterization and finally form the special odor types of different *Rhododendron* varieties.

### 2.4. Odor Analysis

**Figure 4:**
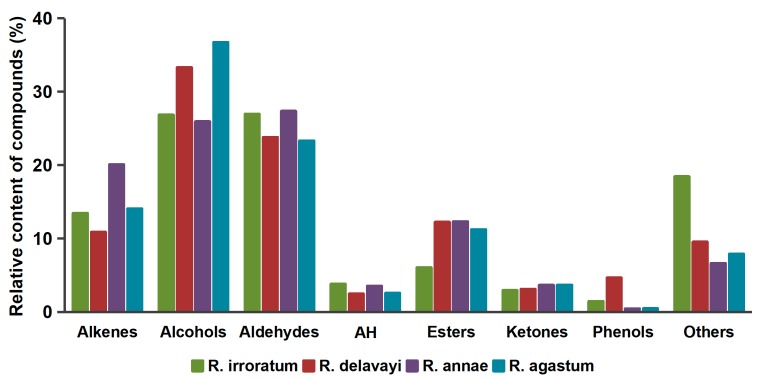
Distribution of the chemical classes for *Rhododendron* (AH: aromatic hydrocarbons).

The identified components were classified into various types of compound groups, including alcohols (29), aldehydes (15), alkenes (29), aromatic hydrocarbons (10), esters (19), ketones (10), phenols (4), and others (13)—eight classes in total. Figure 4 shows the relative contents of the chemical classes in the four samples. The predominant groups were aldehydes and alcohols, followed by esters and alkenes. Large amounts of aldehydes were detected in *R. annae* (27.37%) and *R. irroratum* (26.95%). Although alkenes had the same number of compounds compared to alcohols, their contents were far below those of alcohols. Besides this, *R. irroratum* had the lowest content of esters (6.04%), while the other three species had similar proportions.

#### 2.4.1. Floral and Woody Odor

From an odor perspective, alcohols showed a higher number of compounds with a descriptor of a floral odor. For example, linalool, which is reported to possess a floral and citrus-like aroma [32], was relatively high in the *R. annae* species (4.752% ± 0.114%). Aside from linalool, benzyl alcohol, phenylethyl alcohol, and citronellol are all described as having floral and rose odors. Among them, phenylethyl alcohol is widely used as ingredient for perfumes and produces a rose smell [33]. Citronellol was previously reported as the floral odor compound in lychee juice [34]. Woody odor attributes in *Rhododendron* flowers were mainly associated with alkenes and alcohols. Alkenes showed a higher number of compounds with descriptors of woody and sweet, such as α-pinene (intense woody), β-pinene (dry woody), and α-terpinene (woody, piney), which were previously identified in terebinth fruits [17], but accounted for relatively low contents (0.117% ± 0.056% to 1.456% ± 0.039%) in flowers. Alcohols such as isopulegol and isoborneol also have woody odor characterization and accounted for 1.099% ± 0.091% to 3.328% ± 0.133% in *Rhododendron* flowers, mostly higher than the alkene contents. In addition, β-ionone, well known for its violet odor and described as a complex woody and fruity scent [35], was also found in four flower species.

#### 2.4.2. Green and Fresh Odor

Grass odor is sometimes referred to as a fresh note, and the chemicals with this descriptor are predominantly aldehydes with six to nine carbons and C6 alcohols [36,37]. In *Rhododendron* flowers, hexanal was the major such compound in all samples, mainly contributing to the green and grassy odor. Besides this, 2-hexenal, heptanal, octanal, and benzeneacetaldehyde was also found to contribute to the green and fresh odor [34]. Among them, 2-hexenal and heptanal accounted for relatively high proportions in *R. annae* and *R. agastum*. On the other hand, C6 alcohols such as (*E*)-3-hexen-1-ol and 1-hexanol also yielded a green, fresh, and herbal odor [32]. In addition, β-cadinene, 2-pentyl-furan and formic acid, 2-phenylethyl ester are also related to a green odor.

#### 2.4.3. Sweet and Fruity Odor

In the *Rhododendron* flowers, the compounds contributing to the sweet and fruity odor mainly included aldehydes and alkenes. Among the aldehydes, citronellal (sweet, citrus), decanal (sweet, orange), and undecanal (floral, citrus) all provide a sweet and fruity odor, especially citronellal with its high contents in the four flower species (4.178% ± 0.654% to 7.944% ± 0.225%). Among the alkenes, limonene is a typical sweet and citrus-like odor compound which was previously identified in lychee [32]. α-Ocimene with a fruity aroma was also reported in a previous study [17]. Some alcohols like major compound 2-nonen-1-ol also have a sweet and melon odor. Besides this, it has been previously reported that α-terpineol is one of the major components providing fruity and floral notes in Pu-erh tea [38]. Other compounds, for example, 2-pentyl-furan, reported to have a fruity, green, and earthy odor [39], accounted for a relatively high proportion in *R. irroratum* (up to 1.643% ± 0.290%) among the four flower species studied.

#### 2.4.4. Total Odor Description

**Figure 5:**
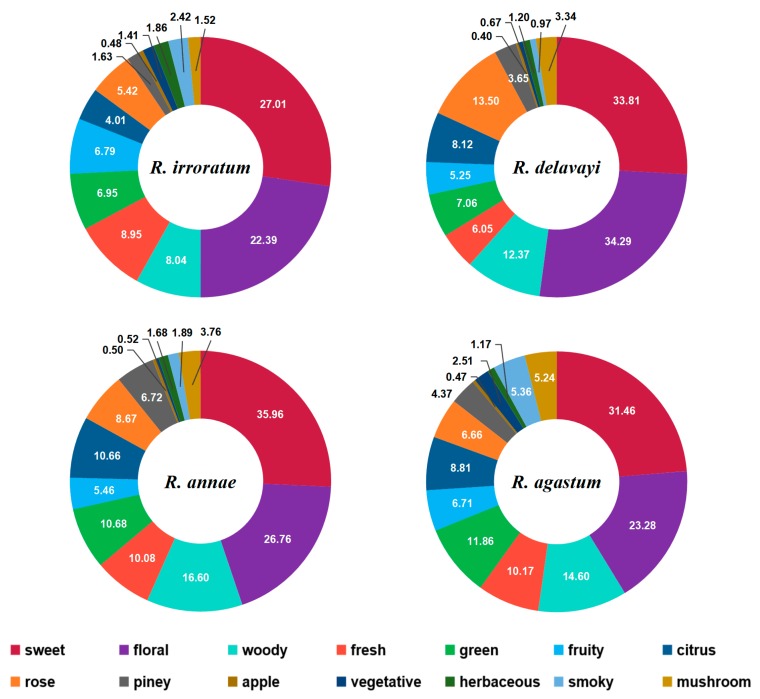
Proportions of odor compounds in *Rhododendron*.

As illustrated by the four pie charts shown in Figure 5, the proportion distributions of volatile compounds based on the specific odor characteristics of the *Rhododendron* flowers were surveyed to represent the odor types of compounds in the samples. There was a higher number of chemical compounds with descriptors of floral, woody, sweet, and fresh odor, mainly derived from alkenes, alcohols, esters, and aldehydes, thus comprising the major odor characteristics of *Rhododendron* flowers. Sweet odor represented the highest proportion in *R. annae* (35.96%), *R. irroratum* (27.01%), and *R. agastum* (31.46%), while floral odor was the most abundant in *R. delavayi* (up to 34.29%). Other odors such as herbaceous, piney, and mushroom had relatively low proportions but also contributed to the overall odor characteristics. The different compounds and contents make up the specific *Rhododendron* odors. Volatile aroma components from various species and their content differences determine the flower-specific scent properties. Their odor values and contributions to flower odorant will be further investigated in the future.

## 3. Materials and Methods

### 3.1. Sample Pretreatment

The flowers from four *Rhododendron* species (*R. delavayi*, *R. agastum*, *R. annae*, and *R. irroratum*) were collected in the spring of 2019 (between March and April) in Baili *Rhododendron* National Forest Reserve (E 105°45’~106°04’ 45"; N 27°08’ 30"~27°20’ 00"), located in northwestern Guizhou, China. Flowers were collected and placed in sealed plastic bags, then immediately transported in a cooler with ice to the laboratory. Subsequently, the obtained samples were smashed after being frozen in a vacuum freeze-dryer for a week at −70 °C (FD-1C-80; Boyikang, Beijing, China), then transferred into 50 mL vials [15]. All samples were stored in a freezer at a temperature below −20 °C until analysis. A mixed sample was prepared using all the four flower species in equal quantities and was used for analytical method establishment and repeatability examination.

### 3.2. SPME Methodology

The extraction and concentration of the volatile compounds were carried out using the headspace solid phase microextraction (HS-SPME) technique. As the object of this study was to characterize all volatile compounds, divinylbenzene/carboxen/polydimethylsiloxane (DVB/CAR/PDMS) fiber (50/30 μm) (Supelco, Bellefonte, PA, USA) combining the characteristics of both carboxen and divinylbenzene adsorbents in the coating and thus allowing a wide range of molecules of different sizes to be adsorbed into the coating for natural products [40], was chosen for volatile compound analysis. Quantities of 50 mg of samples were accurately weighed into a 20 mL vial, and then the SPME fiber was exposed to the headspace of the bottle for 20 min at 70 °C. The SPME fiber was then introduced into the GC injector for 3.0 min to allow thermal desorption of the analytes. The established approach for quantitative analysis was validated by studying the repeatability using the mixed sample. All measurements were conducted in triplicate.

### 3.3. Analytical Instrumentation

The system was equipped with simultaneous 1DGC and GC×GC in one instrument which can conduct both techniques at the same time without any change of columns. The system consisted of a gas chromatograph (7890B Agilent Technology, Santa Clara, CA, USA) coupled with a high-resolution quadrupole time-of-flight mass spectrometer (QTOFMS) (mass resolution 20,000 and a mass accuracy specification of 3 ppm) (7250, Agilent Technology). In the presented research, an HP-5 MS (5% phenyl–95% dimethylpolysiloxane, 30 m × 250 μm, 0.25 μm film) was used as the ^1^D column, and a DB-17 MS column (50% phenyl–50% dimethylpolysiloxane, 1.2 m × 180 μm, 0.18 μm film) was used as the ^2^D column. The samples were introduced by a split/splitless injector (SSL) system with an autosampler (PAL RSI 120, CTC Technologies). This study employed a technique to combine GC×GC and 1DGC components into a single system with the column outlet of each component connected at the same three-port splitter prior to the QTOFMS detection. This allowed direct comparison of the GC×GC and 1DGC results and avoided use of a second detector, which is simple and effective.

The 1DGC and GC×GC conditions were the same and were as listed below: the GC injector was kept at 250 °C in splitless mode; helium (99.999%) was used as the carrier gas at a constant flow of 1.2 mL/min; oven temperature was initially set at 50 °C (held for 3 min) , then increased to 250 °C at 4 °C /min (held for 7 min), for a total run time of 60 min. The GC×GC system was coupled with an SSM1800 solid state modulator (J&X Technologies, China). The GC×GC conditions were as follows: The cold zone temperature of the SSM was set at −50 °C. The temperatures of the entry hot zone and exit hot zone were +30 and +120 °C offset relative to oven temperatures, respectively, with a cap temperature of 320 °C for both hot zones. The modulation period was 4 s.

The MS conditions were as follows: The electron ionization and the ion source and transfer line temperatures were set at 70 eV, 250 °C, and 280 °C, respectively. The MS scan rate was 50 Hz. The mass range was set to 50–500 *m*/*z* in full-scan acquisition mode.

### 3.4. Data Method

The volatile composition was quantified in duplicate by HS-SPME coupled to GC×GC with QTOFMS according to the method of previous reports [41]. The 1DGC data were processed using Agilent Mass Hunter Qualitative Analysis navigator B.08.00; the GC×GC data were analyzed using dedicated Canvas GC×GC data processing software (J&X Technologies, version v1.4.0, Shanghai, China). Tentative compound identification was accomplished by mass spectral match based on the NIST 17 Mass Spectral Library (NIST/EPA/NIH 2017) and then verified using the retention index (RI) and accurate mass. The RI was calculated using a series of *n*-alkanes (C8–C25) analyzed on an HP-5 MS column under the same chromatographic conditions. The odor identification method was performed based on previous studies [42], relying on the Good Scents Company Information System, available online: http://www.thegoodscentscompany.com.

## 4. Conclusions

GC×GC-QTOFMS was applied to identify the volatile aroma compounds in four *Rhododendron* flower species. In total, 129 volatile compounds were separated and confirmed by spectral similarity, exact mass, and retention index. The relative contents of the volatile compounds were profiled for the four species of *Rhododendron* flowers. With the great advantages of the GC×GC technique over traditional 1DGC, this preliminary study improved scientific understanding regarding the volatile components in *Rhododendron* flowers, and the detected compounds could be used to establish the fingerprint signatures of *Rhododendron*.

## Supplementary Materials

The following are available online at https://www.mdpi.com/1420-3049/24/18/3327/s1, Table S1. Complete compound information for identification; Table S2. Compound contents in *Rhododendron* flowers of different species and their odor description.
