# Supplementary material for: Characterization of Volatile Compounds in Four Different Rhododendron Flowers by GC×GC-QTOFMS
Source: Molecules. 2019 Sep 12;24(18):3327. doi: 10.3390/molecules24183327 (PMC6767277; doi:10.3390/molecules24183327)
Supplement: Supplementary file 1 [file molecules-24-03327-s001.pdf]

Supporting information for

Characterization of Volatile Compounds in Four Different *Rhododendron* Flowers by GC×GC-QTOFMS

Chen-Yu Qian [1,2], Wen-Xuan Quan [2], Zhang-Min Xiang [1,*] and Chao-Chan Li [2,*]

[1] Guangdong Provincial Key Laboratory of Emergency Test for Dangerous Chemicals/Guangdong Engineering and Technology Research Center for Ambient Mass Spectrometry, Guangdong Institute of Analysis, Guangzhou 510070, China; qianchenyu94@163.com

[2] Guizhou Provincial Key Laboratory of Mountainous Environmental Protection, Guizhou Normal University, Guiyang 550001, China; wenxuanq@gznu.edu.cn

* Correspondence: xiangzm@live.com (Z.X.); chaochanl@gznu.edu.cn (C.L.)

Table S1. Complete compound information for identification.

	Compounds	1DGC	GC×GC-QTOF-MS									
		RT (min)	Peak I(min)[a]	Peak II(s)[a]	Matching factor		Molecular ion mass (m/z)		Mass accuracy (ppm)	RI_{lib} [c]	RI_{exp} [d]	RI error[e]
					Forward	Reverse	Theoretical	Experimental				
	**Alkenes**											
1	α-Pinene		13.38	1.24	786	866	136.1247	136.1249	0.147	938	938	0
2	3,7-dimethyl-1-Octene		14.65	1.43	723	838	140.156	No[b]	-	963	971	8
3	3,5,5-trimethyl-2-Hexene		14.85	1.50	722	831	126.1403	No	-	981	976	-5
4	β-Pinene		15.52	1.26	834	913	136.1247	136.1257	0.735	-	993	-
5	3-Carene	16.21	16.32	1.34	846	907	136.1247	136.1247	0.000	1013	1014	1
6	α-Terpinene		16.58	1.34	808	843	136.1247	136.1247	0.000	1019	1021	2
7	Limonene	19.63	17.05	1.35	807	837	136.1247	136.125	0.220	1018	1033	15
8	α-Ocimene		17.72	1.31	746	888	136.1247	136.1251	0.294	1050	1050	0
9	(4E)-10-Methyl-4-undecen e		22.38	1.37	794	820	168.1873	No	-	1158	1173	15

10	7-epi-Silphiperfol-5-ene		28.12	1.24	818	848	204.1873	204.1878	0.245	1348	1334	-14
11	Silphinene		28.45	1.23	855	863	204.1873	204.1873	0.000	1346	1344	-2
12	Cyclosativene		29.65	1.28	810	831	204.1873	204.1876	0.147	1380	1380	0
13	Silphiperfol-5-ene		29.92	1.28	857	859	204.1873	204.1874	0.049	1403	1388	-15
14	α-Gurjunene		31.05	1.31	839	861	204.1873	204.1871	-0.098	1411	1423	12
15	Thujopsene-I3		31.05	1.54	771	789	204.1873	No	-	1416	1423	7
16	α-Cedrene		31.25	1.37	854	892	204.1873	204.1874	0.049	1429	1429	0
17	Caryophyllene	31.29	31.38	1.39	910	926	204.1873	204.1873	0.000	1424	1434	10
18	β-Cedrene		31.52	1.40	857	884	204.1873	204.1869	-0.196	1425	1438	13
19	α-Bergamotene	31.61	31.72	1.24	766	872	204.1873	204.1868	-0.245	1439	1444	5
20	Germacrene		31.65	1.36	794	871	204.1873	204.1869	-0.196	1442	1442	0
21	Calarene		31.85	1.39	788	838	204.1873	204.1879	0.294	1435	1448	13
22	Alloaromadendrene	32.21	32.32	1.36	807	849	204.1873	204.1874	0.049	1463	1463	0
23	1-Pentadecene		32.92	1.09	800	810	210.2342	No	-	1488	1481	-7
24	β-Guaiene	33.43	33.25	1.40	736	761	204.1873	204.1871	-0.098	1492	1492	0
25	α-Bulnesene		33.58	1.41	857	897	204.1873	204.1875	0.098	1502	1502	0

26	α-Muurolene	33.34	33.85	1.37	883	899	204.1873	204.1874	0.049	1502	1511	9
27	Selina-3,7(11)-diene		34.32	1.43	748	808	204.1873	204.1876	0.147	1539	1527	-12
28	β-Cadinene		34.52	1.40	885	895	204.1873	204.1872	-0.049	1528	1534	6
29	3-Heptadecene		38.78	1.04	814	850	238.2655	238.2692	1.553	1688	1681	-7
	**Alcohols**											
30	2,3-Butanediol		8.32	1.60	877	754	90.0675	90.066	-1.665	756	-	-
31	(E)-3-Hexen-1-ol		10.45	1.47	852	888	100.0883	100.0884	0.100	857	857	0
32	1-Hexanol		10.85	1.41	802	812	102.1039	No	-	861	869	8
33	1-Octen-3-ol	14.90	15.05	1.40	881	887	128.1196	No	-	981	981	0
34	Benzyl alcohol	17.18	17.38	2.17	914	921	108.057	108.057	0.000	1040	1042	2
35	1-Octanol		18.58	1.40	887	897	130.1352	No	-	1073	1073	0
36	trans-Linalool oxide (furanoid)	19.63	18.78	1.45	879	885	154.1352	No	-	1101	1078	-3
37	Linalool oxide	19.77	19.38	1.47	728	743	142.1352	No	-	1105	1093	15
38	Linalool	20.28	19.78	1.41	916	916	122.0726	154.1352	0.000	1121	1104	3
39	2-Nonen-1-ol		19.85	1.45	885	886	170.1301	142.1356	0.281	1081	1106	1

40	Phenylethyl Alcohol		20.45	2.16	824	838	170.1301	No	-	1078	1122	1
41	(E)-*p*-2,8-Menthadien-1-ol		20.65	1.60	774	782	152.1196	152.1195	-0.066	1127	1127	0
42	Isopulegol A		21.65	1.56	914	916	154.1352	154.1353	0.065	1161	1154	-7
43	Isopulegol B	21.94	22.05	1.56	932	934	154.1352	154.1353	0.065	1149	1165	16
44	Isoborneol	22.33	22.45	1.68	902	905	154.1352	154.1343	-0.584	1160	1175	15
45	*p*-Menthan-3-ol	22.50	22.65	1.49	913	922	156.1509	No	-	1164	1181	17
46	4-Terpineol	22.72	22.85	1.59	830	853	154.1352	154.1357	0.324	1179	1186	7
47	5-Decanol		23.05	1.27	777	807	158.1665	158.1636	-1.834	1175	1191	16
48	α-Terpineol	23.19	23.32	1.63	900	917	154.1352	No	-	1180	1198	18
49	Ethyllinalool		23.32	1.35	814	867	168.1509	No	-	1182	1198	16
50	Myrtenol	23.45	23.58	1.68	756	824	152.1196	152.1193	-0.197	1213	1206	-7
51	2-Hydroxycineol	23.98	24.12	1.81	906	909	170.1301	170.1302	0.059	1228	1221	-7
52	2-phenoxy-Ethanol	24.26	24.45	2.25	911	911	138.0675	138.0677	0.145	1228	1230	2
53	Citronellol	24.37	24.52	1.42	892	897	156.1509	156.1509	0.000	1231	1232	1
54	2,3-Pinanediol		24.52	1.78	808	829	170.1301	170.1302	0.059	1244	1232	-12
55	1-Dodecanol	32.61	32.72	1.28	781	819	186.1978	No	-	1475	1475	0

56	Spathulenol		36.52	1.54	777	801	220.1822	220.1823	0.045	1582	1600	18
57	Cedrol		37.18	1.66	884	892	222.1978	222.199	0.540	1631	1624	-7
58	β-Eudesmol		38.52	1.62	872	905	222.1978	222.1994	0.720	1651	1672	21
	**Aldehydes**											
59	Hexanal		8.58	1.40	917	939	100.0883	100.0881	-0.200	776	802	26
60	2-Hexenal		10.38	1.61	898	914	98.0726	98.0729	0.306	855	855	0
61	Heptanal		12.05	1.47	863	894	114.1039	114.1045	0.526	880	904	24
62	(Z)-2-Heptenal		14.18	1.66	858	930	112.0883	112.0885	0.178	958	959	1
63	Benzaldehyde		14.45	2.19	929	932	106.0413	106.0412	-0.094	965	966	1
64	Octanal	15.84	15.92	1.50	852	924	128.1196	No	-	1004	1004	0
65	4-methyl-3-Cyclohexene-1<br>-carboxaldehyde		17.18	1.83	809	876	124.0883	124.0884	0.081	1024	1037	13
66	Benzeneacetaldehyde	17.54	17.65	2.25	837	855	120.057	120.0579	0.750	1047	1049	2
67	(E)-2-Octenal	18.00	18.12	1.60	850	918	126.1039	126.1042	0.238	1061	1061	0
68	Citronellal	21.64	21.78	1.48	891	900	154.1352	154.1355	0.195	1155	1157	2
69	Lilac aldehyde		22.98	1.98	748	751	168.1145	168.1148	0.178	1169	1190	21

70	Decanal	23.55	23.65	1.40	890	890	156.1509	156.1501	-0.512	1208	1208	0
71	Undecanal	27.15	27.25	1.35	907	935	170.1665	170.1648	-0.999	1308	1308	0
72	Kharismal		38.25	1.75	828	891	226.1563	226.1561	-0.088	1650	1662	12
73	1-Pentadecanal		39.78	1.22	792	915	226.2291	No	-	1717	1717	0
	**Aromatic hydrocarbons**											
74	Toluene		7.85	1.32	791	843	92.0621	92.0621	0.000	757	-	-
75	*p*-Cymenene		19.45	1.63	891	934	132.0934	132.0936	0.151	1092	1095	3
76	Nonylcyclopentane		31.98	1.07	836	860	196.2186	196.219	0.204	1452	1452	0
77	α-Corocalene		31.98	1.43	795	870	200.156	200.1564	0.200	-	1452	-
78	*cis*-Calamenene	34.47	34.58	1.53	844	869	202.1716	202.1716	0.000	1531	1536	5
79	n-Nonylcyclohexane		35.18	1.07	832	856	210.2342	210.2343	0.048	1556	1556	0
80	β-Calacorene		35.25	1.64	872	922	200.156	200.1564	0.200	1560	1558	-2
81	1,2,3,5,6,7-hexahydro-4,8-dimethyl-s-Indacene		36.72	1.76	791	827	186.1403	186.1404	0.054	1639	1608	-31
82	Undecyl cyclopentane		38.18	1.05	863	895	224.2499	224.2501	0.089	1660	1660	0
83	Cadalene		39.12	1.82	880	893	198.1403	198.1403	0.000	1677	1693	16

	**Esters**											
84	Hexanoic acid, methyl ester		12.92	1.44	911	914	130.0988	130.0989	0.077	926	926	0
85	Butanedioic acid, dimethyl ester	17.00	17.12	2.02	907	951	146.0574	No	-	1034	1035	1
86	Benzoic acid, methyl ester		19.72	2.02	795	848	136.0519	136.0526	0.515	-	1102	-
87	Octanoic acid, methyl ester		20.58	1.39	898	907	158.1301	158.1304	0.190	1125	1125	0
88	Pentanedioic acid, dimethyl ester	20.97	21.12	1.93	851	902	160.073	No	-	1138	1140	2
89	benzyl acetate		22.25	2.05	804	888	150.0675	150.0676	0.067	1168	1170	2
90	Formic acid, 2-phenylethyl ester		22.72	2.07	806	952	150.0675	150.0675	0.000	1179	1183	4
91	Methyl salicylate	23.36	23.52	1.98	861	892	152.0468	152.0471	0.197	1194	1204	10
92	Nonanoic acid, methyl ester		24.32	1.33	898	903	172.1458	172.1461	0.174	1226	1226	0
93	Hexanedioic acid, dimethyl ester	24.89	25.05	1.89	884	893	174.0887	No	-	1246	1247	1

94	β-Phenethyl acetate		25.72	1.92	791	801	164.0832	No	-	1261	1265	4
95	Bornyl acetate	26.61	26.72	1.52	889	891	196.1458	196.1461	0.153	1288	1293	5
96	Decanoic acid, methyl ester		27.78	1.31	884	918	186.1614	186.1612	-0.107	1325	1324	-1
97	1,3-Pentanediol, 2,2,4-trimethyl-, 1-isobutyrate	29.57	29.72	1.46	879	888	216.172	No	-	1380	1382	2
98	Dodecanoic acid, methyl ester		34.25	1.23	886	896	214.1927	214.1927	0.000	1525	1525	0
99	Benzoic acid, 2,6-dimethoxy-, methyl ester		34.92	2.78	853	856	196.073	196.0731	0.051	1536	1548	12
100	2,2,4-Trimethyl-1,3-pentanediol diisobutyrate		36.58	1.28	827	855	286.2139	No	-	1591	1603	12
101	Methyl tetradecanoate		40.05	1.20	867	886	242.224	242.2246	0.248	1710	1727	17
102	Hexadecanoic acid, methyl ester		45.32	1.15	906	910	270.2553	270.2556	0.111	1927	1927	0
	**Ketones**											

103	methyl heptenone		15.32	1.62	827	897	126.1039	126.1039	0.000	988	988	0
104	dihydro-4,4-dimethyl-2,3-Furandione		16.25	2.32	713	754	128.0468	128.0468	0.000	-	1013	-
105	(E)-3-Octen-2-one	17.25	17.38	1.55	764	888	126.1039	126.104	0.079	1036	1042	6
106	(E,E).octa-3,5-dien-2-one	18.51	18.65	1.78	829	903	124.0883	124.088	-0.242	1073	1074	1
107	Camphor		21.65	1.85	739	805	152.1196	152.1202	0.394	1146	1154	8
108	Dihydropseudoionone	31.99	32.12	1.47	897	910	194.1665	194.1666	0.052	1457	1457	0
109	2,6-Di-tert-butyl-4-hydroxy-4-methylcyclohexa-2,5-dien-1-one		32.78	1.38	840	854	236.1771	236.1779	0.339	1477	1477	0
110	1,3,4,7-Tetramethyltricyclo[5.3.1.0(4,11)]undec-2-en-8-one		33.12	1.40	783	826	218.1665	218.1666	0.046	-	1488	-
111	β-Ionone		33.32	1.65	794	842	192.1509	192.1515	0.312	1493	1494	1
112	Benzophenone		37.78	2.45	921	930	182.0726	182.0727	0.055	1645	1646	1
	**Phenols**											
113	Phenol		15.45	1.91	828	929	94.0413	94.0416	0.319	984	992	8

114	4-ethyl Phenol	22.27	22.45	1.91	875	898	122.0726	122.0729	0.246	1172	1175	3
115	4-ethyl-2-methoxy Phenol		26.45	1.90	837	871	152.0832	152.0833	0.066	1286	1286	0
116	2,4,6-Triisopropyl phenol	34.00	34.12	1.51	852	855	220.1822	220.1853	1.408	1516	1520	4
	**Others**											
117	Pyridine	7.12	7.32	1.61	856	890	79.0417	79.0418	0.127	731	-	-
118	2-methyl-Butanoic acid		10.25	1.34	759	867	102.0675	No	-	841	851	10
119	2-pentyl-Furan	15.41	15.52	1.39	880	900	138.1039	138.1041	0.145	993	993	0
120	1,2-dimethoxybenzene	21.39	21.52	2.20	877	903	138.0675	138.0676	0.072	1151	1151	0
121	1-adamantyl methyl ether		23.18	1.34	806	842	166.1352	166.1358	0.361	1178	1195	17
122	3,5-Dimethoxy toluene	25.86	25.98	1.99	849	850	152.0832	No	-	1273	1273	0
123	4-ethyl-1,2-dimethoxy-Benzene		27.92	1.95	886	887	166.0988	166.0989	0.060	1326	1328	2
124	1,2-Diacetin		28.65	1.90	871	890	176.0679	No	-	-	1350	-
125	1,2,3-trimethoxy-5-methyl-Benzene	30.43	30.58	2.09	929	930	182.0937	182.0958	1.153	1409	1409	0
126	1-Pentadecyne		33.92	1.28	795	876	208.2186	208.2177	-0.432	1514	1514	0

127	Elemicin	35.32	1.98	841	933	208.1094	208.1097	0.144	1558	1561	3
128	Diethyltoluamide	36.18	2.11	892	909	191.1305	191.1298	-0.366	1571	1590	19
129	Isoelemicin	38.18	2.02	818	841	208.1094	208.1099	0.240	1654	1660	6

[a] Retention times in minutes (min) for first (Peak I) dimensions and seconds (s) for second (Peak II) dimensions.

[b] No—undetected molecular ion in the mass profile.

[c] Retention index from https://webbook.nist.gov/chemistry.

[d] Retention index from the present study measured on an HP-5MS column against a mixture of C8–C25 hydrocarbons.

[e] Error = Calculated RI − Literature RI. The Literature I values and the corresponding citations are shown in Table S1 in the Supporting information.

Table S2. Compound contents in *Rhododendron* flowers of different species and their odor description.

No.	Compounds	Relative content (%)				Intraday RSD(%)	Interday RSD(%)	Odor description[a]
		R. irroratum	R. delavayi	R. annae	R. agastum			
1	Pyridine	2.209 ±0.298	0.967 ±0.006	1.805 ±0.115	5.357 ±0.140	16.30	18.49	Burnt, coffee
2	Toluene	1.059 ±0.123	0.518 ±0.010	0.949 ±0.015	0.484 ±0.049	1.89	12.59	Sweet

3	2,3-Butanediol	0.140±0.033	0.454±0.009	0.218±0.007	0.104±0.016	9.07	6.35	Fruity
4	Hexanal	6.648±1.199	2.227±0.067	3.706±0.001	3.720±0.423	0.41	12.58	Grassy,green
5	2-methyl-Butanoic acid	0.141±0.003	1.014±0.156	0.372±0.004	0.072±0.054	3.32	4.55	Fruity, acidic
6	2-Hexenal	2.265±0.115	1.123±0.034	2.765±0.392	3.683±0.180	1.56	11.16	Sweet almond, fruity
7	(E)-3-Hexen-1-ol	0.099±0.005	0.468±0.008	0.426±0.008	0.374±0.007	4.23	2.32	Green, leafy
8	1-Hexanol	0.558±0.050	0.651±0.026	0.879±0.005	0.652±0.027	11.28	10.48	Green, fresh, herbal
9	Heptanal	1.153±0.042	0.804±0.036	2.264±0.019	2.016±0.033	1.32	21.15	Fresh, green, citrus odor
10	Hexanoic acid, methyl ester	0.093±0.009	0.055±0.013	0.050±0.001	-	1.20	14.27	Fruity
11	α-Pinene	0.522±0.054	0.373±0.002	0.592±0.017	0.447±0.024	14.80	12.36	Intense woody
12	(Z)-2-Heptenal	0.274±0.064	0.117±0.005	0.177±0.002	0.128±0.024	11.34	22.48	
13	Benzaldehyde	1.173±0.011	1.281±0.011	2.334±0.023	0.743±0.015	13.21	16.63	Sweet, almond, cherry
14	3,7-dimethyl-1-Octene	0.094±0.008	0.133±0.007	0.239±0.001	0.117±0.005	2.32	4.06	Woody, piney
15	3,5,5-trimethyl-2-Hexene	0.108±0.007	0.111±0.005	0.026±0.000	0.206±0.004	5.69	0.40	
16	1-Octen-3-ol	1.224±0.257	0.674±0.009	0.277±0.342	2.512±0.203	16.17	1.89	Mushroom, fungal
17	methyl heptenone	0.489±0.020	0.410±0.010	0.522±0.031	0.472±0.020	7.01	4.41	Fruity, apple
18	Phenol	0.150±0.013	0.445±0.004	-	0.028±0.006	4.69	5.48	Phenolic

19	2-pentyl-Furan	1.643±0.290	0.234±0.013	0.270±0.007	0.364±0.103	1.53	3.47	Fruity, green
20	β-Pinene	0.117±0.056	0.429±0.01	0.641±0.015	0.505±0.027	5.56	20.10	Dry-woody, fresh-pine
21	Octanal	0.633±0.029	1.049±0.142	0.796±0.047	0.436±0.072	4.42	7.73	Green,citrus
22	dihydro-4,4-dimethyl-2,3-Furandi one	0.353±0.004	0.096±0.005	0.206±0.023	0.925±0.011	4.88	11.66	
23	3-Carene	1.083±0.105	1.208±0.021	1.596±0.026	1.371±0.051	3.20	10.99	Sweet, diffusive
24	α-Terpinene	0.130±0.008	0.118±0.005	1.456±0.039	0.112±0.017	7.80	10.36	Woody, piney, citrus
25	Limonene	3.856±0.125	3.269±0.034	4.136±0.125	3.917±0.095	10.05	11.81	Citrus, orange, sweet
26	Butanedioic acid, dimethyl ester	0.415±0.023	0.490±0.001	0.444±0.018	0.505±0.014	8.68	23.64	Sweet fruity, green floral
27	4-methyl-3-Cyclohexene-1-carbox aldehyde	0.296±0.051	0.053±0.001	0.049±0.003	0.056±0.018	16.33	7.79	
28	(E)-3-Octen-2-one	0.303±0.057	0.204±0.011	0.244±0.016	0.153±0.028	10.66	2.88	
29	Benzyl alcohol	0.521±0.041	0.783±0.025	3.760±0.096	1.179±0.054	14.14	22.26	Floral, rose
30	Benzeneacetaldehyde	6.255±0.951	7.013±0.059	6.349±0.062	2.987±0.357	0.16	5.07	Sweet floral, hyacinth
31	α-Ocimene	0.147±0.006	0.108±0.007	0.087±0.001	0.138±0.005	3.43	5.00	Fruity, floral
32	(E)-2-Octenal	0.739±0.142	0.268±0.006	0.582±0.020	0.245±0.056	1.51	16.14	Fresh cucumber, green

33	1-Octanol	0.914±0.064	0.745±0.085	1.059±0.049	1.179±0.066	18.33	0.59	
34	(E,E).octa-3,5-dien-2-one	0.309±0.039	0.123±0.003	0.102±0.001	0.256±0.015	15.66	0.63	Fruity, green grassy
35	trans-Linalool oxide (furanoid)	-	1.028±0.031	-	0.033±0.010	12.37	2.89	Floral
36	Linalool oxide	0.521±0.026	0.476±0.003	0.429±0.005	0.613±0.012	11.45	6.67	Floral, woody, fruity
37	*p*-Cymenene	0.790±0.114	0.405±0.002	0.554±0.013	0.400±0.043	4.05	10.45	Herbaceous
38	Linalool	1.315±0.155	1.262±0.016	4.752±0.114	2.362±0.095	14.54	5.57	Citrus, floral, sweet
39	Benzoic acid, methyl ester	0.052±0.012	0.076±0.002	0.092±0.002	0.066±0.006	18.42	7.87	Wintergreen, almond and floral
40	2-Nonen-1-ol	4.299±0.288	5.633±0.813	1.092±0.034	4.071±0.378	7.08	1.33	Sweet,melon
41	Phenylethyl Alcohol	2.336±0.319	8.922±0.061	2.413±0.200	2.129±0.193	5.30	13.67	Floral, dried rose flower
42	Octanoic acid, methyl ester	0.129±0.003	0.349±0.059	0.145±0.001	0.129±0.021	2.63	0.11	Fruity, citrus
43	(E)-*p*-2,8-Menthadien-1-ol	0.190±0.015	0.215±0.006	0.267±0.013	0.114±0.011	7.15	14.80	Fresh minty
44	Pentanedioic acid, dimethyl ester	1.575±0.096	3.002±0.096	1.566±0.089	1.679±0.094	3.64	1.12	Floral
45	1,2-dimethoxybenzene	0.283±0.023	0.819±0.007	0.069±0.014	0.147±0.014	2.14	8.81	Sweet creamy, vanilla
46	Isopulegol A	3.392±0.814	-	-	7.722±0.407	4.73	7.89	
47	Camphor	0.433±0.068	1.115±0.043	0.833±0.086	0.769±0.066	12.10	15.11	Intense spicy smell of camphor
48	Citronellal	4.178±0.654	7.944±0.225	7.004±0.028	7.722±0.303	10.06	8.90	Sweet, floral

49	Isopulegol B	2.744±0.125	3.328±0.133	3.080±0.026	3.053±0.095	8.53	10.48	Minty, woody
50	benzyl acetate	0.010±0.003	0.050±0.007	0.032±0.010	0.040±0.007	16.64	19.84	Sweet, fruity and floral
51	(4E)-10-Methyl-4-undecene	0.337±0.001	0.245±0.089	0.369±0.008	0.294±0.033	0.43	1.45	
52	Isoborneol	1.099±0.091	2.440±0.043	1.453±0.025	2.360±0.053	8.41	9.48	Balsam, herbal, woody
53	4-ethyl Phenol	0.020±0.000	3.482±0.041	0.287±0.011	0.205±0.018	3.07	11.10	Phenolic castoreum
54	*p*-Menthan-3-ol	1.003±0.136	0.902±0.021	1.192±0.026	1.530±0.061	10.99	1.55	Peppermint, cool woody
55	4-Terpineol	0.032±0.002	0.027±0.001	0.494±0.036	0.036±0.013	0.03	1.74	Pepper woody, sweet
56	Formic acid, 2-phenylethyl ester	0.308±0.025	0.474±0.01	0.053±0.002	0.455±0.012	12.50	15.75	Rose, green hyacinth
57	Lilac aldehyde	2.999±0.342	0.738±0.016	0.052±0.019	0.598±0.125	10.43	3.32	Sweet, flowery
58	5-Decanol	-	-	0.098±0.000	0.480±0.000	8.89	1.56	
59	1-adamantyl methyl ether	0.142±0.000	0.131±0.004	-	0.078±0.002	0.62	0.95	
60	α-Terpineol	0.141±0.000	0.020±0.003	0.207±0.033	0.025±0.012	7.50	11.86	Pine, lilac, citrus
61	Ethyllinalool	0.780±0.140	0.709±0.023	0.607±0.000	2.012±0.054	1.16	3.15	Fresh, floral, rich
62	Myrtenol	0.225±0.025	0.285±0.004	0.331±0.015	0.312±0.015	9.54	0.94	Woody, pine, mint
63	Methyl salicylate	0.120±0.002	0.083±0.000	0.194±0.008	0.215±0.004	5.17	12.31	Sweet, with aromatic and balsamic nuances

64	Decanal	0.624±0.213	0.729±0.029	0.973±0.007	0.516±0.083	8.93	15.73	Sweet, orange peel, floral
65	2-Hydroxycineol	1.019±0.092	0.256±0.003	0.061±0.000	0.561±0.032	6.47	1.16	
66	Nonanoic acid, methyl ester	0.127±0.015	0.114±0.004	0.155±0.006	0.197±0.008	18.75	4.36	Sweet, fruity, pear
67	2-phenoxy-Ethanol	0.995±0.076	1.168±0.022	0.775±0.000	1.024±0.032	5.59	13.33	Mild rose
68	Citronellol	0.821±0.274	2.032±0.171	1.14±0.012	1.533±0.152	9.86	4.41	Floral, rose, citrus
69	2,3-Pinanediol	0.214±0.010	0.163±0.010	0.161±0.000	0.383±0.007	10.96	6.16	
70	Hexanedioic acid, dimethyl ester	1.268±0.055	1.112±0.080	0.557±0.013	0.678±0.049	9.34	6.78	Mild nutty
71	β-Phenethyl acetate	0.078±0.011	0.171±0.009	0.033±0.013	0.051±0.011	3.39	8.08	Floral, rose, honey, fruity
72	3,5-Dimethoxytoluene	7.052±1.518	4.172±0.102	2.981±0.101	0.690±0.574	5.69	17.44	
73	4-ethyl-2-methoxy-Phenol	0.022±0.001	0.630±0.027	-	0.084±0.014	7.54	2.04	Spicy, bacon
74	Bornyl acetate	1.292±0.425	2.525±0.201	4.122±0.392	3.294±0.339	3.59	1.36	Woody, pine, cedar spice
75	Undecanal	0.262±0.010	0.235±0.001	0.292±0.010	0.275±0.007	14.22	1.62	Floral, citrus, fresh
76	Decanoic acid, methyl ester	0.059±0.005	0.111±0.058	0.100±0.002	0.091±0.022	15.24	4.55	Oily, fruity
77	4-ethyl-1,2-dimethoxy-Benzene	0.047±0.011	0.489±0.014	0.015±0.006	0.032±0.010	1.72	2.04	Musty earthy
78	7-epi-Silphiperfol-5-ene	0.179±0.004	0.155±0.037	0.638±0.004	0.203±0.015	0.15	0.60	
79	Silphinene	-	0.218±0.033	1.067±0.003	0.372±0.012	8.87	8.25	

80	1,2-Diacetin	0.021±0.002	0.234±0.047	0.174±0.001	0.122±0.017	11.78	4.74	
81	Cyclosativene	0.272±0.014	0.131±0.019	0.252±0.008	0.277±0.014	2.17	20.76	
82	1,3-Pentanediol, 2,2,4-trimethyl-, 1-isobutyrate	0.164±0.024	0.297±0.017	0.355±0.002	0.323±0.014	14.94	20.02	
83	Silphiperfol-5-ene	0.857±0.056	0.644±0.239	2.399±0.005	1.051±0.100	9.47	4.31	
84	1,2,3-trimethoxy-5-methyl-Benzene	6.046±0.623	0.678±0.036	0.243±0.023	0.250±0.227	3.98	3.34	
85	α-Gurjunene	0.409±0.027	0.355±0.004	0.52±0.014	0.349±0.015	7.83	6.62	Woody, balsam
86	Thujopsene-I3	0.057±0.067	0.036±0.001	0.256±0.017	0.052±0.028	1.82	14.40	
87	α-Cedrene	0.218±0.009	0.203±0.002	0.244±0.005	0.223±0.005	6.39	7.37	Woody, cedar, sweet, fresh
88	Caryophyllene	-	-	1.277±0.009	0.827±0.009	4.84	9.45	Sweet, woody, clove
89	β-Cedrene	0.638±0.052	0.719±0.013	-	0.110±0.033	4.90	4.85	
90	α-Bergamotene	-	0.059±0.001	0.162±0.031	0.114±0.011	11.96	2.76	
91	Germacrene	0.257±0.075	0.176±0.02	0.269±0.004	0.213±0.033	7.36	15.28	
92	Calarene	0.130±0.004	0.050±0.002	0.440±0.008	0.079±0.005	3.66	4.23	Woody
93	Nonylcyclopentane	0.535±0.045	0.412±0.013	0.493±0.032	0.433±0.030	6.92	3.76	

94	α-Corocalene	0.130±0.004	0.050±0.002	0.440±0.008	0.021±0.005	11.32	1.80	
95	Dihydropseudoionone	0.242±0.041	0.132±0.014	0.423±0.017	0.288±0.024	8.08	4.74	Fresh rose leaf, floral
96	Alloaromadendrene	0.228±0.018	0.165±0.008	0.367±0.003	0.280±0.010	6.12	0.35	Woody
97	1-Dodecanol	0.163±0.009	0.005±0.001	0.004±0.000	-	12.66	3.94	Honey, coconut
98	2,6-Di-tert-butyl-4-hydroxy-4-methylcyclohexa-2,5-dien-1-one	0.105±0.040	0.222±0.004	0.219±0.053	0.268±0.032	4.71	6.53	
99	1-Pentadecene	1.159±0.336	1.070±0.060	1.149±0.024	1.197±0.140	3.98	17.67	
100	1,3,4,7-Tetramethyltricyclo[5.3.1.0(4,11)]undec-2-en-8-one	0.262±0.0220	0.199±0.006	0.197±0.002	0.037±0.010	2.41	2.64	
101	β-Guaiene	0.826±0.039	0.490±0.013	0.750±0.005	0.593±0.019	1.73	3.20	Sweet, woody
102	β-Ionone	0.131±0.107	0.335±0.006	0.692±0.038	0.241±0.051	24.70	1.91	Floral, woody, sweet, berry
103	α-Bulnesene	0.332±0.025	0.066±0.001	0.064±0.002	0.063±0.009	6.11	0.20	
104	α-Muurolene	0.630±0.089	0.180±0.005	0.486±0.009	0.368±0.034	1.10	8.87	
105	1-Pentadecyne	0.595±0.040	0.412±0.007	0.029±0.000	0.300±0.016	0.58	14.09	
106	2,4,6-Triisopropylphenol	1.671±0.586	0.153±0.003	0.183±0.009	0.169±0.199	0.45	1.50	
107	Dodecanoic acid, methyl ester	0.137±0.031	2.608±0.090	3.183±0.076	2.724±0.066	2.52	12.28	Creamy, coconut, mushroom

108	Selina-3,7(11)-diene	0.145±0.034	0.201±0.019	0.201±0.013	0.211±0.022	11.75	1.97	
109	β-Cadinene	0.468±0.114	0.180±0.012	0.395±0.041	0.215±0.056	6.81	9.16	Green, woody
110	*cis*-Calamenene	0.448±0.074	0.411±0.018	0.364±0.063	0.730±0.052	4.01	3.53	
111	Benzoic acid, 2,6-dimethoxy-, methyl ester	0.041±0.039	0.258±0.022	0.840±0.019	0.436±0.027	7.71	19.35	
112	n-Nonylcyclohexane	0.211±0.074	0.188±0.001	-	0.006±0.038	10.93	10.87	
113	β-Calacorene	0.172±0.081	0.322±0.015	0.409±0.020	0.349±0.039	12.12	8.24	
114	Elemicin	0.109±0.018	0.060±0.004	0.106±0.004	0.117±0.009	5.79	4.02	Spice, flower
115	Diethyltoluamide	0.046±0.014	0.012±0.000	0.009±0.000	0.016±0.005	9.81	2.90	
116	Spathulenol	0.023±0.000	0.037±0.001	0.054±0.003	0.010±0.001	9.69	11.84	Honey
117	2,2,4-Trimethyl-1,3-pentanediol diisobutyrate	0.050±0.001	0.089±0.000	0.151±0.007	0.081±0.002	4.15	0.60	
118	1,2,3,5,6,7-hexahydro-4,8-dimethyl-s-Indacene	0.153±0.042	0.050±0.011	0.143±0.013	0.034±0.022	11.64	10.56	
119	Cedrol	0.241±0.009	0.233±0.002	0.258±0.003	0.234±0.005	0.41	1.20	
120	Benzophenone	0.279±0.036	0.339±0.022	0.302±0.001	0.284±0.019	6.12	1.96	Balsam, rose, geranium
121	Isoelemicin	0.193±0.050	0.368±0.018	0.380±0.019	0.342±0.029	9.20	3.00	

122	Undecylcyclopentane	0.430 ±0.122	0.013 ±0.003	0.022 ±0.000	0.013 ±0.041	12.04	5.18	
123	Kharismal	0.064 ±0.011	0.059 ±0.006	0.069 ±0.008	0.075 ±0.008	3.80	10.01	Floral, jasmine, green
124	β-Eudesmol	0.064 ±0.001	0.086 ±0.012	0.099 ±0.012	0.138 ±0.008	0.88	7.46	Woody, green
125	3-Heptadecene	0.154 ±0.002	0.150 ±0.001	0.155 ±0.017	0.158 ±0.007	10.91	9.84	
126	Cadalene	0.093 ±0.003	0.088 ±0.014	0.097 ±0.007	0.110 ±0.008	10.11	5.71	
127	1-Pentadecanal	0.094 ±0.001	0.070 ±0.013	0.174 ±0.008	0.122 ±0.008	14.39	7.29	Fresh, waxy
128	Methyl tetradecanoate	0.131 ±0.004	0.126 ±0.002	0.134 ±0.004	0.138 ±0.003	1.26	14.64	Petal
129	Hexadecanoic acid, methyl ester	0.127 ±0.016	0.117 ±0.006	0.253 ±0.045	0.135 ±0.022	16.96	9.07	Orris

[a] Description according to http://www.thegoodscentscompany.com.
